# Predictors of Postoperative Epilepsy/Seizures in Patients Readmitted After Meningioma Resection

**DOI:** 10.1155/bn/5717503

**Published:** 2025-07-09

**Authors:** Rasha Elbadry, Anthony O. Asemota, Brandon Edelbach, Lei Huang, Firas Bannout, Warren Boling

**Affiliations:** ^1^Department of Neurosurgery, Loma Linda University Medical Center, Loma Linda, California, USA; ^2^Department of Basic Science, Loma Linda University, Loma Linda, California, USA; ^3^Department of Neurology, Loma Linda University Medical Center, Loma Linda, California, USA

**Keywords:** meningioma, new-onset epilepsy/seizure electrocorticography, peritumoral edema, postoperative epilepsy/seizures, readmissions

## Abstract

**Background:** Epilepsy/seizures in meningioma patients may occur pre- or postoperatively, causing significant morbidity and impaired quality of life. Surgical excision is considered a standard management with variable rates of epilepsy/seizure resolution reported after surgery. Employing a national database, we examined the pre- and postoperative incidences of epilepsy/seizures and risk factors associated with postoperative epilepsy/seizures in patients readmitted within 30 days and/or 90 days following meningioma resection.

**Methods:** The 2010–2014 Nationwide Readmissions Database was analyzed. Consecutive patients undergoing surgery for meningioma resection were identified using appropriate ICD-9-CM codes. Standard descriptive techniques and multivariate regression were used to identify predictors of postoperative epilepsy/seizure after discharge.

**Results:** Among 46,107 patients undergoing meningioma resection at index hospitalization, 20.40% (*n* = 9408) had preoperative epilepsy/seizure diagnosis. The mean patient age was 58.37 ± 13.85 years. Patients with preoperative epilepsy/seizures were more likely to be male (*p* < 0.001), frail (*p* < 0.001), and with higher comorbidity index scores (*p* < 0.001). The overall readmission rate was 30.36% and was higher among patients with preoperative epilepsy/seizures (36.66% vs. 28.75%, *p* < 0.001). Respectively, 30- and 90-day readmission rates were higher among patients (13.22% vs. 11.73%, *p* < 0.001) and (23.25% vs 20.30%, *p* = 0.04) with epilepsy/seizure diagnosis at index admission. Predictors of postoperative epilepsy/seizures at 30- and 90-day readmissions included the preoperative epilepsy/seizure, malignant meningioma, peritumoral cerebral edema, and higher comorbidity index scores, while male sex was significant only at 30-day readmissions. Intraoperative electrocorticography was associated with a decreased likelihood of postoperative epilepsy/seizures.

**Conclusion:** Development of epilepsy/seizures after meningioma resection is likely multifactorial. Identifying factors associated with postoperative epilepsy/seizures after discharge is important in triaging and closer monitoring of at-risk patients and for adapting management to help improve outcomes.

## 1. Introduction

Meningiomas are intracranial tumors arising from meningothelial cells of the dura coverings of the brain. They are usually well-encapsulated, slow-growing, and often clinically silent, discovered incidentally during brain imaging for some other condition. Otherwise, they may cause symptoms from mass effect on adjacent brain structures. They generally show a female predominance and are usually located along the brain convexity, parasagittal location, and skull base[1]. According to the World Health Organization, meningiomas are classified into Grades I–III based on severity and histological appearance [2]. Grade I, which is considered benign, is the most common and least severe; Grade II is atypical, while Grade III is anaplastic or malignant meningioma and considered the most aggressive type. Surgical excision is the standard management for meningiomas, particularly for symptomatic patients and individuals with large or rapidly expanding tumors [1, 3, 4].

Epilepsy/seizures are symptoms commonly encountered among individuals with intracranial meningiomas along with headaches and neurologic deficits [1, 5, 6]. They may occur pre- or postoperatively, causing significant morbidity and impaired quality of life [1, 5–9]. Surgical excision has been reported to result in complete resolution of symptoms in variable proportions of patients; however, others continue to experience symptoms despite tumor excision [6, 10, 11]. Epilepsy/seizure onset might also occasionally follow tumor resection [12]. Employing a national database, we examine incidence rates of recurrent and new-onset epilepsy/seizures among patients readmitted within 30 days and 90 days of discharge. Additionally, we examine predictors of postoperative epilepsy/seizures associated with readmissions after meningioma resection.

## 2. Methods

This study was conducted in accordance with the STROBE guidelines.

### 2.1. Data Source

Data was obtained from the Nationwide Readmissions Database (NRD) 2010–2014 (“NRD Overview,” n.d.). The NRD includes discharge records for patients with and without repeat hospital visits in a year and those who have died in the hospital, regardless of the expected payer for the hospital stay. The NRD includes records from approximately 18 million discharges yearly when unweighted and estimates approximately 35 million discharges when weighted. As the NRD is a publicly available database, institutional review board approval was not required for this study.

### 2.2. Inclusion Criteria

NRD search inclusion criteria included the following: (1) adult patients, (2) diagnosis of intracranial meningioma, and (3) surgical resection of meningioma. Patients less than 18 years old were excluded from data. Diagnosis of intracranial meningioma was identified using ICD-9-CM diagnosis codes (225.2, 192.1, and 237.6). Patients undergoing meningioma resection identified using ICD-9-CM procedural codes were included for the study (Table S1). Incomplete records, defined as absent clinical variables for 30-day follow-up, were excluded. Patient records were queried and cross-referenced for consistency in diagnoses, procedures, and postoperative outcomes. Additionally, postoperative epilepsy/seizures occurring after index discharge identified using ICD-9-CM diagnosis codes and verified via query of patient records (Table S1).

### 2.3. Definition of Terms

*Index admission/hospitalization* is the admission during which patient underwent meningioma surgery.

*Index discharge* is the discharge date from index admission/hospitalization.

*Readmissions* are any subsequent admission succeeding the index admission/hospitalization.

*30-day readmissions* are readmissions occurring within 30 days of index discharge.

*90-day readmissions* are readmissions occurring within 90 days of index discharge.

*Preoperative epilepsy/seizure* is present in the patient's index hospitalization record.

*Postoperative epilepsy/seizure* is present on any subsequent hospitalization record after index discharge.

### 2.4. Variables Included

Demographic variables included patient age, sex, frailty status, comorbidities, insurance payer status, and median household income. Hospital-level variables included hospital teaching status, location, and bed size. Charlson Comorbidity Index (CCI) was employed in grouping patient comorbidities [13]. Frailty was characterized using a previously validated algorithm for national administrative data [14]. Tumor variables included meningioma type/character classified as benign (corresponding to WHO Grade I), malignant (corresponding to WHO Grade III), and uncertain. Patient records were queried to ascertain the presence of peritumoral cerebral edema, radiological evidence of brain compression, hydrocephalus, intracerebral hemorrhage, fluid-electrolyte changes, central nervous system (CNS) infection (i.e., bacterial meningitis), and steroid use. *Intraoperative* use of electrocorticography and electroencephalographic (EEG) monitoring was identified using appropriate ICD-9-CM procedure codes [15] (Table S1).

### 2.5. Primary Outcomes

The main outcome was postoperative epilepsy/seizures occurring after index discharge identified using ICD-9-CM diagnosis codes (Table S1). We calculated incidence rates and identified predictors of postoperative epilepsy/seizures. Additional outcomes were length of stay obtained directly from the database, calculated for both index cases and readmissions.

### 2.6. Statistical Analyses

**Figure 1:**
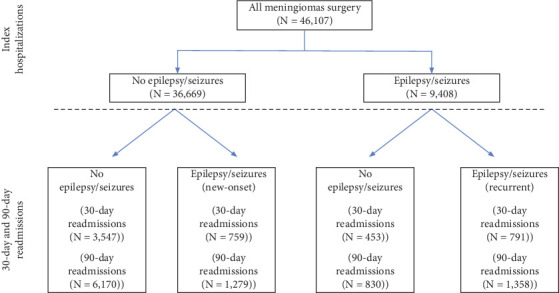
Flow chart outlining study plan.

All statistical analyses were performed using Stata 17.0 (StataCorp). Standard weights contained within the NRD were employed in obtaining population level counts and proportions. Analyses were performed across index admissions and readmissions. With respect to index admissions, univariate chi-squared analysis examined all variables of interest in relation to outcome. Two main cohorts were identified based on the presence of preoperative epilepsy/seizure diagnosis as “epilepsy” and “no-epilepsy” cohorts and analyses involved comparisons between cohorts (Figure 1). Listwise deletion was utilized to handle missing values in the “epilepsy” and “no-epilepsy” cohorts. Separate analyses were performed to examine 30-day and 90-day readmissions. As with index cases, univariate associations examined variables across readmissions with respect to postoperative epilepsy/seizures. Multivariate analyses examined potential predictors of postoperative seizures for 30-day readmissions and 90-day readmissions in separate models. For multivariate analyses, covariates with *p*-values less than 0.20 in bivariate analyses were used to build a final model using the backward elimination technique. Model sensitivity and goodness of fit were assessed using the Hosmer–Lemeshow goodness-of-fit tests. All analyses were performed at significance level of *p* < 0.05 and results presented in tables/figures.

*Subset analyses* were performed among patients with intractable epilepsy and status epilepticus. Interactions between patient age and gender, as well as between peritumoral cerebral edema and brain compression, were examined in separate analyses.

### 2.7. Sensitivity Analyses

To minimize the influence of sampling differences across years, unweighted data was analyzed, and results were compared with weighted data.

## 3. Results

### 3.1. Univariate Analyses

#### 3.1.1. Index Hospitalizations

A total of 46,107 patients underwent surgery for meningioma resection during index admission, 20.40% (9408 patients) of whom had a preoperative epilepsy/seizure diagnosis. The mean age of patients who underwent surgery was 58.37 ± 13.85 years.

There were significant differences noted between study arms in our cohort. Majority of patients were less than 65 years old (65.36%); however, patients with epilepsy compared to no epilepsy had greater proportions of ≥ 65-year olds (36.47% vs. 34.17%, *p* = 0.02). Patients with epilepsy/seizures were more likely male (41.12% vs. 28.58%, *p* < 0.001), have higher CCI scores (*p* < 0.001), belong to lower median income quartile groups (*p* < 0.001) and to possess government-sponsored insurance or to be uninsured, and were less likely to have private insurance (*p* < 0.001). Frailty, present in 7.20%, was more common in patients with epilepsy (9.35% vs. 6.65%, *p* < 0.001). Surgeries were mostly performed at metropolitan-teaching hospitals (83.27%) and at large bed size hospitals (79.38%) (Table 1).

**Figure 2:**
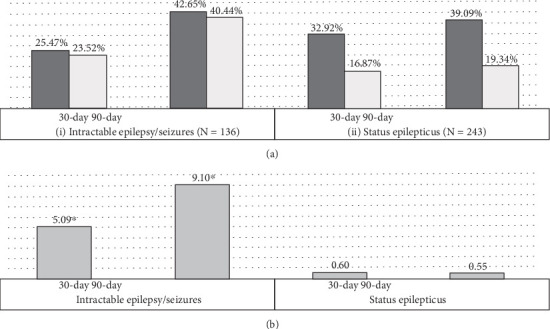
Cases of intractable epilepsy/seizures and status epilepticus. (a) Readmissions as proportions of index cases: (i) for patients with intractable epilepsy, index cases = 136, 30 − day readmissions = 25.47%, 30 − day epilepsy/seizure recurrence = 23.52%, 90 − day readmissions = 42.65%, 90-day epilepsy/seizure recurrence = 40.44%. (ii) For patients with status epilepticus, index cases = 243, 30 − day readmissions = 32.92%, 30 − day epilepsy/seizure recurrence = 16.87%, 90 − day readmissions = 39.09%, 90-day epilepsy/seizure recurrence = 19.34%. (b) Odds ratios of postoperative epilepsy/seizure diagnosis present on readmission: (i) for patients with intractable epilepsy/seizures, 30-day readmissions (OR = 5.09; 95%CI = 1.07–24.22; *p* < 0.001), 90-day readmissions (OR = 9.10; 95%CI = 1.96–42.17; *p* < 0.001). (ii) For patients with status epilepticus, 30-day readmissions (OR = 0.60; 95%CI = 0.20–1.80; *p* = 0.36), and 90-day readmissions (OR = 0.55; 95%CI = 0.20–1.48; *p* = 0.23). The asterisk indicates statistically significant difference.

Epilepsy compared to no-epilepsy patients were more commonly diagnosed with malignant (4.92% vs. 3.30%) and uncertain (1.92% vs. 1.00%) and less commonly benign (93.15% vs. 95.70%) meningiomas (*p* < 0.001). Similarly, epilepsy patients were commonly diagnosed with peritumoral cerebral edema (40.07% vs. 24.98%, *p* < 0.001) and intracerebral hemorrhage (4.20% vs. 2.25%, *p* < 0.001). There were no significant differences between epilepsy and no-epilepsy cohorts in the proportions of patients with hydrocephalus (*p* = 0.08), radiologic evidence of brain compression (*p* = 0.39), or steroid use (*p* = 0.62). There were no significant differences between epilepsy and no-epilepsy patients in the use of intraoperative electrocorticography (1.51% vs. 1.27%, *p* = 0.25); however, epilepsy patients were more likely to have undergone EEG monitoring (5.02% vs. 0.99%, *p* < 0.001) (Table 2). Among patients with epilepsy/seizures, a diagnosis of intractable epilepsy was mentioned in 1.44%, and status epilepticus occurred in 2.58% (Figure 2).

#### 3.1.2. Readmissions

The overall readmission rate was 30.36% and was higher among patients with epilepsy/seizures at index admission (36.66% vs. 28.75%, *p* < 0.001). Out of 5550 readmissions that occurred within 30 days, 27.93% (1550 cases) had a diagnosis of epilepsy/seizures. Out of 9637 readmissions that occurred within 90 days, 27.36% (2637 cases) had a diagnosis of epilepsy/seizures (Figure 1).

For patients with epilepsy/seizure diagnosis at index admission, the 30-day and 90-day readmission rates were, respectively, 13.22% (1244 cases) and 23.26% (2188 cases). For patients without epilepsy/seizure diagnosis at index admission, the 30-day and 90-day readmission rates were, respectively, 11.73% (4306 cases) and 20.30% (7449 cases). Overall, 30-day (13.22% vs. 11.73%, *p* < 0.001) and 90-day (23.25% vs. 20.30%, *p* < 0.001) readmission rates were higher among patients having epilepsy/seizure diagnosis at index admission.

##### 3.1.2.1. Recurrent Versus New-Onset Epilepsy/Seizures

Recurrent epilepsy/seizure: Of 9408 patients with epilepsy/seizure diagnosis at index admission, 8.41% (791 patients) were readmitted with epilepsy/seizure recurrence within 30 days and 14.43% (1358 patients) within 90 days of index discharges (Figure 1).

New-onset epilepsy/seizures: Of 36,699 patients without epilepsy/seizure diagnosis at index admission, 2.07% (759 cases) and 3.49% (1279 cases) were, respectively, readmitted with new-onset epilepsy/seizure diagnosis within 30 days and 90 days of index discharges (Figure 1).

##### 3.1.2.2. 30-Day and 90-Day Readmissions

The overall rates of postoperative epilepsy/seizure diagnoses, whether new-onset or recurrent, were 27.93% (1550/5550 cases) for 30-day readmissions and 27.36% (2637/9637 cases) for 90-day readmissions. Univariate comparisons of 30-day readmissions revealed significant differences between epilepsy and no-epilepsy cohorts by gender (*p* < 0.001), CCI (*p* = 0.01), and peritumoral cerebral edema (*p* = 0.01). There were no significant differences by age group (*p* = 0.62) or frailty (*p* = 0.96). Results are shown in Table 3.

**Figure 3:**
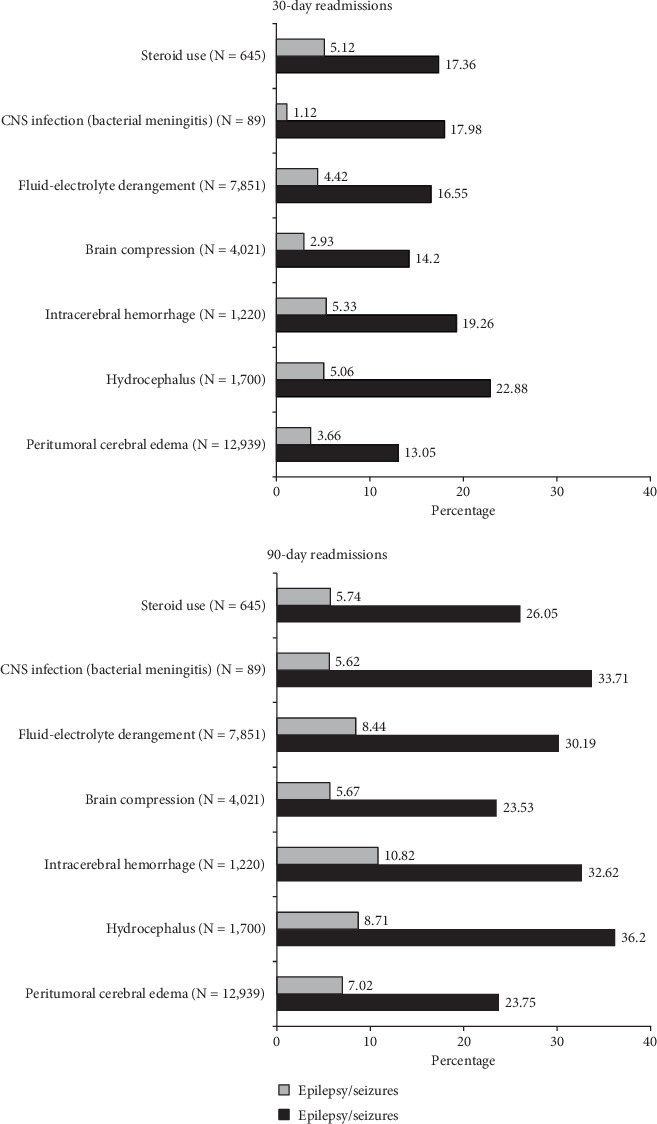
Readmissions as a proportion of index cases. Peritumoral edema: 12,939 index cases, 30-day readmissions = 13.05%, 30-day epilepsy/seizure readmissions = 3.66%; 90-day readmissions = 23.75%, 90-day epilepsy/seizure readmissions = 7.02%. Hydrocephalus: 12,939 index cases: 30-day readmissions = 22.88%, 30-day epilepsy/seizure readmissions = 5.06%; 90-day readmissions = 36.29%, 90-day epilepsy/seizure readmissions = 8.71%. Intracerebral hemorrhage: 1220 index cases, 30-day readmissions = 19.26%, 30-day epilepsy/seizure readmissions = 5.33%; 90-day readmissions = 32.62%, 90-day epilepsy/seizure readmissions = 10.82%. Brain compression: 4021 index cases, 30-day readmissions = 14.20%, 30-day epilepsy/seizure readmissions = 2.93%; 90-day readmissions = 23.53%, 90-day epilepsy/seizure readmissions = 5.67%. Fluid-electrolyte derangement: 7851 index cases, 30-day readmissions = 16.55%, 30-day epilepsy/seizure readmissions = 4.42%; 90-day readmissions = 30.19%, 90-day epilepsy/seizure readmissions = 8.44%. Central nervous system infection, that is, bacterial meningitis: 89 index cases, 30-day readmissions = 17.98%, 30-day epilepsy/seizure readmissions = 1.12%; 90-day readmissions = 33.71%, 90-day epilepsy/seizure readmissions = 5.62%. Steroid use: 645 index cases, 30-day readmissions = 17.36%, 30-day epilepsy/seizure readmissions = 5.12%; 90-day readmissions = 26.05%, 90-day epilepsy/seizure readmissions = 5.74%.

Univariate assessments of 90-day readmissions revealed significant differences between epilepsy and no-epilepsy cohorts by gender (*p* = 0.04), CCI (*p* < 0.001), peritumoral cerebral edema (*p* < 0.001), and intracerebral hemorrhage (*p* = 0.02). There were no significant differences by age group (*p* = 0.08) or frailty (*p* = 0.42). Results are shown in Table 4. The proportions of patients readmitted within 30 and 90 days of index discharge based on tumor and clinical characteristics at index admission are shown in Figure 3.

#### 3.1.3. Duration From Index Discharge to Readmission

The overall mean duration from index discharge to readmissions for 30-day readmissions was 12.30 ± 8.61 days. Patients with epilepsy/seizures at 30-day readmissions had a mean duration from index discharge to readmission of 11.52 ± 8.59 days. Cases of new-onset epilepsy/seizures demonstrated shorter duration from index discharge to readmission compared to recurrent cases (10.65 vs. 12.39 days, *p* = 0.01). For 90-day readmissions, the overall mean duration from index discharge to readmissions was 30.29 ± 25.03 days. Patients with epilepsy/seizures at 90-day readmissions had mean duration from index discharge to readmission of 30.53 ± 26.03 days. There was no significant difference in mean duration from index discharge to readmission comparing new-onset and recurrent cases of epilepsy/seizures (29.06 vs. 31.94 days, *p* = 0.06) (Table 5).

### 3.2. Multivariate Analyses

Multivariate analyses of postoperative epilepsy/seizure diagnosis within 30 days of index discharge revealed the following significant associations: male sex (OR = 1.58; 95%CI = 1.16–2.15; *p* < 0.001), preoperative epilepsy/seizure diagnosis (OR = 8.15; 95%CI = 5.81–11.43; *p* < 0.001), malignant meningioma (OR = 1.74; 95%CI = 1.12–2.69; *p* = 0.01), peritumoral cerebral edema (OR = 1.46; 95%CI = 1.02–2.10; *p* = 0.04), CCI index-1 (OR = 1.56; 95%CI = 1.14–2.13; *p* = 0.01) and CCI score-2 (OR = 1.41; 95%CI = 1.04–1.92; *p* = 0.03), and intraoperative electrocorticography (OR = 0.31; 95%CI = 0.11–0.85; *p* = 0.02). There was no significant association noted with age ≥ 65 (*p* = 0.30), frailty (*p* = 0.67), fluid-electrolyte derangement (*p* = 0.05), brain compression (*p* = 0.22), hydrocephalus (*p* = 0.87), intracerebral hemorrhage (*p* = 0.50), bacterial meningitis (*p* = 0.18), or steroid use (*p* = 0.25) (Table 6).

Multivariate analyses of postoperative epilepsy/seizure diagnosis within 90 days of index discharge revealed the following significant associations: preoperative epilepsy/seizure diagnosis (OR = 8.36; 95%CI = 6.52–10.73; *p* < 0.001), malignant meningioma (OR = 1.90; 95%CI = 1.39–2.61; *p* < 0.001), peritumoral cerebral edema (OR = 1.78; 95%CI = 1.34–2.37; *p* < 0.001), and CCI score-1 (OR = 1.35; 95%CI = 1.06–1.72; *p* = 0.02) and CCI score-2 (OR = 1.28; 95%CI = 1.02–1.59; *p* = 0.03), and intraoperative electrocorticography (OR = 0.21; 95%CI = 0.09–0.48; *p* < 0.001). There was no significant association seen with age ≥ 65 (*p* = 0.77), male sex (*p* = 0.10), frailty (*p* = 0.60), brain compression (*p* = 0.52), hydrocephalus (*p* = 0.76), intracerebral hemorrhage (*p* = 0.74), fluid-electrolyte derangement (*p* = 0.94), bacterial meningitis (*p* = 0.22), or steroid use (*p* = 0.25) (Table 6).

#### 3.2.1. Subset Analyses

**Figure 4:**
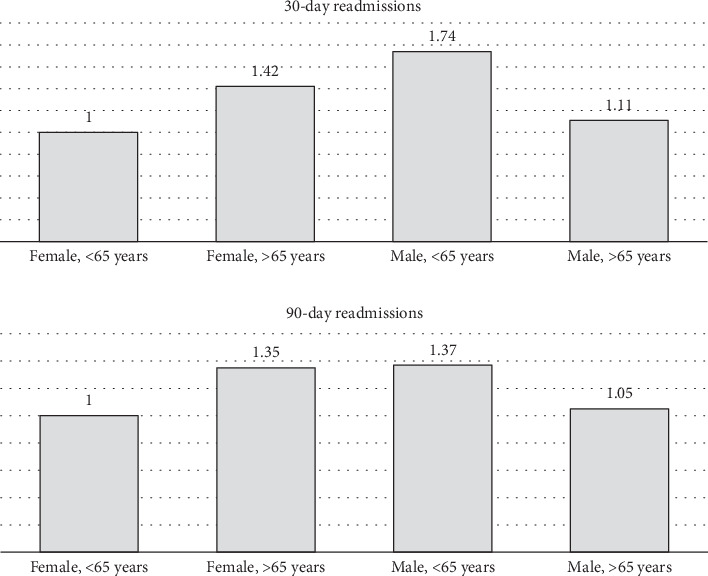
Examining the interaction between age and sex and odds ratios of postoperative epilepsy/seizure diagnosis present on readmission. For 30-day readmissions: females < 65 years (comparison group), females ≥ 65 years (OR = 1.42; 95%CI = 0.88–2.27; *p* = 0.15), males < 65 years (OR = 1.74; 95%CI = 1.24–2.45; *p* < 0.001), males ≥ 65 years (OR = 1.11; 95%CI = 0.68–1.81; *p* = 0.68). For 90-day readmissions: females < 65 years (comparison group), females ≥ 65 years (OR = 1.35; 95%CI = 0.99–2.43; *p* = 0.12), males < 65 years (OR = 1.37; 95%CI = 0.98–1.78; *p* = 0.32), males ≥ 65 years (OR = 1.05; 95%CI = 0.76–1.60; *p* = 0.80).

**Figure 5:**
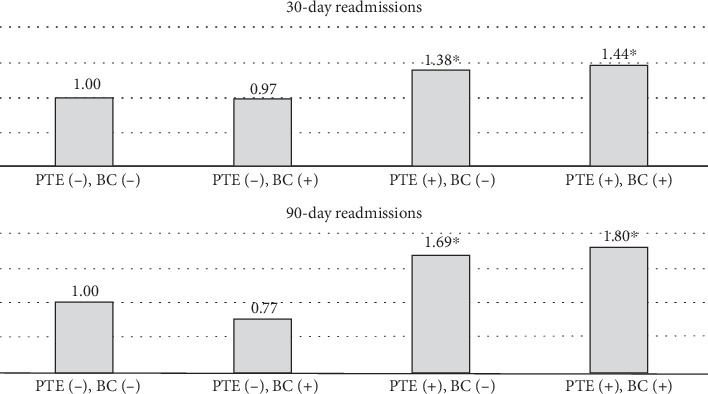
Examining the interaction between peritumoral cerebral edema (PTE) and brain compression (BC) and odds ratios of postoperative epilepsy/seizure diagnosis present on readmission. For 30-day readmissions, PTE−/BC− (comparison group), PTE−/BC+ (OR = 0.97; 95%CI = 0.24–3.93; *p* = 0.15), PTE+/BC− (OR = 1.38; 95%CI = 1.06–1.98; *p* = 0.01), PTE+/BC+ (OR = 1.44; 95%CI = 1.04–2.25; *p* = 0.02). For 90-day readmissions, PTE−/BC− (comparison group), PTE−/BC+ (OR = 0.77; 95%CI = 0.33–1.79; *p* = 0.55), PTE+/BC− (OR = 1.69; 95%CI = 1.26–2.27; *p* < 0.001), PTE+/BC+ (OR = 1.80; 95%CI = 1.06–3.37; *p* = 0.02). The asterisk indicates statistically significant difference.

Among patients with intractable epilepsy revealed significantly higher likelihood of postoperative epilepsy/seizure diagnosis at 30-day and 90-day intervals. No increased likelihood of postoperative epilepsy/seizures was noted in patients with status epilepticus (Figure 2b). Examining the interaction between age and gender revealed male patients < 65 years old were more likely to be readmitted at 30-day postdischarge interval. Analyses of the interaction between brain compression and peritumoral edema showed increased likelihood of postoperative epilepsy/seizures associated with peritumoral edema in the presence and/or absence of brain compression (Figures 4 and 5).

#### 3.2.2. Sensitivity Analyses

Results comparable to our main analyses were obtained when unweighted data were analyzed.

## 4. Discussion

In this study, we examined the incidence of preoperative epilepsy/seizures among patients undergoing craniotomy for intracranial meningioma resection and examined postoperative epilepsy/seizures rates among patients readmitted within 30 and 90 days postdischarge. We evaluated clinicodemographic characteristics associated with preoperative epilepsy/seizures at index admissions as predictors of postoperative epilepsy/seizures within 30 and 90 days of index discharge. Overall, epilepsy/seizures were diagnosed preoperatively in ~20.40% of patients who underwent surgery and postoperatively in ~27.93% of 30-day readmissions and ~27.36% of 90-day readmissions after index discharge. After adjustment for confounders, several predictors of postoperative epilepsy/seizures were identified among readmitted patients including the presence of preoperative epilepsy/seizure, tumor type (malignant), peritumoral cerebral edema, greater patient comorbidity, male sex, and intraoperative use of electrocorticography.

To the knowledge of the authors, this is the first retrospective study examining postoperative epilepsy/seizures among nationwide readmitted patients after meningioma surgery and identifying potential predictors.

### 4.1. Predictors of Epilepsy/Seizure After Surgery

We found preoperative epilepsy/seizures as highly predictive of postoperative epilepsy/seizure in patients readmitted within 30 and 90 days of index discharge. Similar finding of greater postoperative epilepsy/seizure risk after meningioma resection among patients with preoperative epilepsy/seizure has been reported by a number of studies [10, 16–19]. Specifically, postoperative epilepsy/seizure risk was higher among patients diagnosed with intractable epilepsy/seizures on index admission, accounting for approximately 92%–95% of readmissions among this subgroup of patients. Interestingly, status epilepticus present on index admission did not correlate with an increased risk of postoperative epilepsy/seizures in 30- or 90-day readmissions. Although not directly examined in this study, prior studies have reported a significant correlation of postoperative epilepsy/seizures with the duration and severity of preoperative epilepsy/seizures [17], suggesting a possible benefit in prioritizing early surgery for patients with preoperative epilepsy/seizures.

Results from our study revealed significant association between malignant meningioma—corresponding to WHO Grade III classification—and increased risk of postoperative epilepsy/seizures within 30 and 90 days of index discharge. Prior studies have also reported higher rates of postoperative epilepsy/seizures with malignant and higher grade meningiomas [18, 20]. A number of factors might be linked to epilepsy/seizure occurrence with malignant meningioma including brain invasion and associated gliotic reaction seen with malignant meningiomas, along with dissemination of tumor cells and neurotoxic proteins [1, 21]. The underlying tumor genomics, for example, neurofibromatosis Type 2 (NF2)–gene mutation associated with more aggressive behavior of atypical and higher grade meningioma, might also correlate with an increased predisposition to seizures [22]. Additionally, the increased likelihood of incomplete or subtotal resection and higher rates of recurrence, both more expected with malignant meningiomas [22, 23], could potentially exacerbate persistent epileptogenic activity postoperatively.

Our findings reveal significant associations between peritumoral cerebral edema and higher epilepsy/seizure rates preoperatively in index admissions as well as postoperatively in 30- and 90-day readmissions. Adjusted multivariate analyses showed peritumoral cerebral edema at index admission predicted postoperative epilepsy/seizures within 30 and 90 days postdischarge. Prior studies have also reported the association of peritumoral edema and postoperative epilepsy/seizure risk following meningioma resection [24, 25]. Several pathophysiological mechanisms might account for epilepsy/seizure association with peritumoral cerebral edema. The edema accumulation and progression could lead to disruption of the cortical layer separating tumor from white matter with resultant denervation hypersensitivity [5, 26]. The edema fluid mostly vasogenic in origin and imbued with neurotransmitters could lead to peritumoral changes and trigger neuronal hyperexcitability, ultimately lowering seizure threshold [5, 26]. Furthermore, the extent of edema and involvement of surrounding sensitive foci likely influenced by meningioma histological characteristics [22, 27] could in turn impact totality of tumor resection and recurrence, thus increasing the odds of postoperative epilepsy/seizure occurrence [27–29].

The risk of overall postoperative epilepsy/seizures among readmitted patients was higher with greater patient comorbidity. Compared to patients without comorbidities, patients having one or more comorbidities were more likely to present with postoperative epilepsy/seizures in 30 or 90 days postdischarge. Several factors might explain this association. The polypharmacy in patients with higher comorbidity could complicate epilepsy/seizure management and hypothetically increase their risk of seizures [30]. Alternatively, ongoing epilepsy and recurrent seizures might worsen brain damage exposing sufferers to greater physical and psychological comorbidity [30, 31]. Given higher comorbidity associated with older age, likewise, frailty is more prevalent in the elderly [32, 33]. In our analyses, there was no significant independent postoperative epilepsy/seizure risk based on frailty after adjustment for multiple confounders. With respect to patient age, although a higher incidence of preoperative epilepsy/seizure was seen among individuals over 65 years old in unadjusted analyses, we did not find any significant association between older patient age and postoperative epilepsy/seizures after adjustment in multivariate analyses of 30-day and 90-day readmissions. Further research on the relationship between patient age, comorbidity, and frailty and their influence on outcome after meningioma surgery would be important.

Consistent with the literature, our study revealed higher rates of preoperative epilepsy/seizures among male patients [7]. Findings on multivariate analyses demonstrated an overall increased postoperative epilepsy/seizure risk most notably within 30 days of index discharge. Further exploration revealed a pattern of elevated epilepsy/seizure risk specifically among younger male patients. Admittedly, the incidence of epilepsy/seizures has been demonstrated across multiple studies to be higher among males [16, 24]. Interestingly, also, male sex has been shown to be associated with an increased risk for higher grade meningioma [34]. Although the exact reasons are unknown, sex-hormonal differences and gender-specific patterns of receptor expression seen with higher meningioma grades and known to predict meningioma recurrence [35, 36] could also potentially impact epilepsy/seizure threshold and likely postoperative occurrence. While these assumptions are speculative, further research would be helpful to better understand these associations.

In our analysis, the use of intraoperative electrocorticography correlated with decreased rates of postoperative epilepsy/seizure diagnosis within 30 days and/or 90 days. Consistent with our findings, intraoperative electrocorticography has previously been shown to predict lower incidence of early postoperative epileptiform discharges in patients undergoing meningioma resection; however, no obvious benefit was seen with late-onset epileptogenic activity [37]. Besides its role for intraoperative functional mapping and epileptogenic localization during meningioma resection, electrocorticography might have additional benefits for confirming the extent of resection of epileptogenic foci after surgery.

Our study found no significant independent association of postoperative epilepsy/seizure risk with fluid-electrolyte derangement, brain compression, hydrocephalus, intracerebral hemorrhage, CNS infection (meningitis), or steroid use. Although some of these factors have previously been associated with postoperative epilepsy/seizures in prior studies [11, 12], it is reasonable that continued progression and/or new development of postoperative epilepsy/seizures involves a combination of factors.

### 4.2. Epilepsy/Seizure Resolution/Control After Surgery

There are at present no clinical trials evaluating the effectiveness of surgery in controlling postoperative meningioma-related epilepsy/seizures. Currently available data has relied on retrospective studies reporting variable rates of postoperative epilepsy/seizure incidence after meningioma resection [10, 11, 17, 25, 38, 39]. Many of these studies however involve single centers and limited number of providers. While 85%–90% of patients with preoperative epilepsy will not have seizure recurrence, our study highlights the continued burden of epilepsy/seizures among readmitted patients—approximately 3 in 10 readmitted patients (i.e., 27.93% in 30 days and 27.36% in 90 days) were diagnosed with postoperative epilepsy/seizures. Our results show that among patients with preoperative epilepsy/seizure on index admissions, the diagnosis of seizure recurrence increases overtime, with postoperative epilepsy/seizure recurrence rates of ~8.41% at 30 days and ~14.43% at 90 days of index discharge. Of 36,699 patients without epilepsy/seizure diagnosis at index admission, rates of new-onset epilepsy/seizures increased from ~2.07% to ~3.49% within 30 days and 90 days of index discharge. Taken together, this represents a postoperative epilepsy/seizure readmission rate of ~3.36% and ~5.72%, respectively, within 30 days and 90 days of index discharge regardless of preoperative epilepsy/seizure status. The rates of postoperative epilepsy/seizure recurrence and new-onset epilepsy reported in our study are comparable to previously published findings [7, 18, 24, 25]. However, these observed rates must be interpreted cautiously as they might underrepresent actual postoperative epilepsy/seizure rates since the NRD includes only readmissions and does not capture patients who did not get readmitted and/or individuals that may have presented only at outpatient settings. Furthermore, as tumor location data was not available to us, we were unable to stratify risk of postoperative epilepsy/seizures by location. Nonetheless, our estimates provide a useful baseline for quantifying the burden of postoperative epilepsy/seizures after meningioma resection and by extension assessing the effectiveness of surgery in epilepsy/seizure control.

Regarding the clinical utility of these predictive variables, the utilization of AEDs prophylactically for epilepsy/seizure control perioperatively and after discharge following meningioma surgery is controversial [7, 40]. There is a lack of consensus on its effectiveness, and studies reporting AED use show that practice patterns differ widely [6]. While potential benefits of AEDs might be more readily apparent for patients with preoperative epilepsy/seizures, special consideration might be necessary for seizure-naïve patients who although not having ongoing seizures subsequently develop new-onset epilepsy/seizures after meningioma surgery. Investigation of the efficacy of AED prophylaxis is hindered by the low frequency of postoperative seizures, resulting in significant Type II error. Consequently, most studies investigating the therapeutic efficacy of prophylactic AED are unable to make significant conclusions [41–43]. This limitation imposed by the Type II error could be mitigated by selecting for a cohort with increased risk of postoperative seizure. Recently, a grading criteria based on brain parenchyma invasion, intranuclear inclusion bodies, and use of > 2 antiepileptics for preoperative seizure control was proposed by Singh et al.; however, the study population utilized to formulate this guideline was limited (*n* = 333) [44]. Additionally, Skardelly et al. also analyzed preoperative risk factors and found that nonskull base tumor, tumor volume greater than 8 cm^3^, and male gender were associated with greater risk [45]. Thus, in addition to the variable proposed by Singh et al. and Skardelly et al., our results complement and supplement these prior stratifications [44, 45]. Namely, in addition to increased risk found among males and preoperative epilepsy/seizures, high-grade meningiomas and peritumoral cerebral edema further delineate those with increased risk for postoperative seizure incidence.

From our perspective, given that nearly half (~49%) of all readmissions with postoperative epilepsy/seizure diagnoses occurred in seizure-naïve patients, identifying predisposing factors helpful for patient selection and recommendations based on identified factors would be important. While we found several risk factors including male gender, peritumoral cerebral edema, and high histological grade associated with postoperative epilepsy/seizures in our study, future research is necessary to provide definitive selection criteria for seizure-naïve patients at increased risk of postoperative epilepsy/seizures. Additionally, prospective studies following high-risk patients are necessary to delineate the postoperative history of these patients, prior to assessing the impact of AED in high-risk cohorts. In our study, due to the lack of reliable indicators of patients treated with AEDs within the NRD, we are unable to assess their impact on postoperative epilepsy/seizures after meningioma resection.

### 4.3. Study Limitations

The NRD reports readmissions and follows patients over 12 months after index discharges (“NRD Overview,” n.d.). Therefore, events occurring beyond the follow-up period are not captured in the database, potentially leading to an underestimation of readmissions. This is unlikely to significantly impact our study since we focus on events occurring within 30 and 90 days of index discharges. It is possible that some events considered preoperative might have occurred intra- or postoperatively given a lack of specific identifiers within the database to enable an accurate distinction in all cases. This could potentially lead to exaggerated rates of preoperative events encountered in index cases. While we believe there is minimal skewness in preoperative counts, our designation of postoperative events occurring after index discharges is however unlikely to be affected. Moreover, since our sample included only inpatients who underwent surgery and not all inpatients diagnosed with meningiomas, our reporting of incidence and/or preoperative rates should be interpreted cautiously.

Intraoperative metrics not examined in this study including extent of resection and degree of retraction might influence outcome and likelihood of postoperative epilepsy/seizures following meningioma surgery. Also not examined was tumor location/lateralization previously reported to predict postoperative epilepsy/seizures [16, 17, 39]. These unexamined variables may represent biomarkers permitting quantification into the postoperative risk of epilepsy/seizures and are potential avenues of research. In contrast to preoperative seizures being more likely with cerebral convexity or parasagittal meningiomas, postoperative epilepsy/seizures are reportedly more likely with skull base meningiomas [16, 39]. The absences of reliable indicators within our database limit evaluation of these parameters. Regarding epilepsy and seizure diagnosis, we are unable to evaluate seizure duration or frequency in all instances given lack of specific qualifiers in our database. Consistent with recommendations from the International League Against Epilepsy, distinguishing “epilepsy” separate from “seizure” would necessarily account for these metrics [46]. Hence, from a practical standpoint, in our analyses, we elected as our outcome any diagnosis of “epilepsy/seizure” occurrence.

This study is observational and retrospective in nature and as such subject to inherent biases [47–50]. Since we utilize administrative data, there is the potential for miscoding and entry errors into the database from source. However, this effect is largely minimized given the large volume of cases analyzed. Additionally, as listwise deletion was utilized for missing primary outcome data, we acknowledge that this may introduce bias as patients in the “no-epilepsy” cohort likely possessed reduced incentive for postdischarge monitoring as compared to their symptomatic counterparts. Despite these limitations, our study is unique in that it involves vast number of cases across multiple healthcare settings and providers significantly enhancing its generalizability across diverse patient populations. Additional strengths derive from our analyses employing a longitudinal database which allows patient follow-up across time thus facilitating ample assessment of events leading to readmissions among patients.

## 5. Conclusion

Postoperative epilepsy/seizure after meningioma surgery constitutes a significant burden among readmitted patients. Predictors of postoperative epilepsy/seizures in patients readmitted within 30 days and/or 90 days after meningioma surgery include preoperative epilepsy/seizures, malignant meningioma, peritumoral cerebral edema, and patient comorbidity, as well as male sex in 30-day readmissions only. Intraoperative electrocorticographic monitoring is associated with decreased rates of postoperative seizures/epilepsy among readmitted patients. Higher rates of postoperative epilepsy/seizures occur in patients with an index diagnosis of intractable epilepsy; however, there was no increased risk noted for patients with status epilepticus. The development of epilepsy/seizures after meningioma resection is likely multifactorial. Quantifying the burden of hospital readmissions related to postoperative epilepsy/seizure occurrence after meningioma surgery provides a baseline for appraising early success of surgery and management approaches. Understanding factors associated with postoperative epilepsy/seizures after meningioma surgery is important for identifying patients who are most at risk and adapting management to help improve overall patient outcomes.

## Figures and Tables

**Table 1:** Patient and hospital characteristics for index admissions/hospitalizations.

**Index hospitalizations**	**No epilepsy**		**Epilepsy**		**Total**		**p** **-value**
	**36,699**	**(100.00)**	**9408**	**(100.00)**	**46,107**	**(100.00)**	
Age group							0.02
< 65 years	24,158	(65.83)	5977	(63.53)	30,135	(65.36)	
≥ 65 years	12,541	(34.17)	3431	(36.47)	15,972	(34.64)	
Gender							< 0.001
Male	10,488	(28.58)	3868	(41.12)	14,357	(31.14)	
Female	26,211	(71.42)	5539	(58.88)	31,750	(68.86)	
Charlson Comorbidity Index (CCI)							< 0.001
None	21,385	(58.27)	4392	(46.68)	25,776	(55.90)	
Score 1	7635	(20.80)	1930	(20.51)	9566	(20.75)	
Score ≥ 2	7679	(20.92)	3086	(32.80)	10,765	(23.35)	
Frailty							< 0.001
No	34,260	(93.35)	8528	(90.65)	42,788	(92.80)	
Yes	2439	(6.65)	880	(9.35)	3319	(7.20)	
Median household income							< 0.001
1st quartile (< $40,000)	8186	(22.31)	2396	(25.47)	10,582	(22.95)	
2nd quartile ($40,000–$49,999)	8671	(23.63)	2213	(23.52)	10,884	(23.61)	
3rd quartile ($50,000–$64,999)	9077	(24.73)	2338	(24.85)	11,415	(24.76)	
4th quartile (≥ $65,000)	10,144	(27.64)	2260	(24.02)	12,403	(26.90)	
Unknown	622	(1.69)	201	(2.14)	823	(1.78)	
Insurance coverage							< 0.001
Government	15,994	(43.58)	4856	(51.62)	20,850	(45.22)	
Private	18,069	(49.24)	3769	(40.06)	21,838	(47.36)	
No insurance	2636	(7.18)	783	(8.32)	3419	(7.42)	
Hospital location, teaching status							0.01
Metropolitan, nonteaching	5523	(15.05)	1604	(17.05)	7128	(15.46)	
Metropolitan, teaching	30,732	(83.74)	7659	(81.41)	38,391	(83.27)	
Nonmetropolitan	444	(1.21)	145	(1.54)	588	(1.28)	
Hospital bed size							0.99
Small	1576	(4.29)	405	(4.30)	1981	(4.30)	
Moderate	5999	(16.35)	1529	(16.25)	7527	(16.33)	
Large	29,124	(79.36)	7474	(79.44)	36,598	(79.38)	

**Table 2:** Tumor and clinical characteristics for index admissions/hospitalizations.

**Index hospitalizations**	**No epilepsy**		**Epilepsy**		**Total**		**p** **-value**
	**36,699**	**(100.00)**	**9408**	**(100.00)**	**46,107**	**(100.00)**	
Meningioma type/behavior							< 0.001
Benign	35,121	(95.70)	8764	(93.15)	43,885	(95.18)	
Malignant	1211	(3.30)	463	(4.92)	1674	(3.63)	
Uncertain	367	(1.00)	181	(1.92)	547	(1.19)	
Peritumoral cerebral edema	9169	(24.98)	3770	(40.07)	12,939	(28.06)	< 0.001
Hydrocephalus	1402	(3.82)	298	(3.17)	1700	(3.69)	0.08
Intracerebral hemorrhage	825	(2.25)	395	(4.20)	1220	(2.65)	< 0.001
Brain compression	3236	(8.82)	785	(8.34)	4021	(8.72)	0.39
Fluid-electrolyte derangement	5664	(15.43)	2187	(23.24)	7851	(27.03)	< 0.001
CNS infection (bacterial meningitis)	68	(0.19)	21	(0.22)	89	(0.19)	0.59
Steroid use	505	(1.38)	140	(1.49)	645	(1.40)	0.62
Intraoperative electromonitoring	821	(2.24)	613	(6.52)	1434	(3.11)	< 0.001
Electrocorticography (ECoG)	465	(1.27)	142	(1.51)	608	(1.32)	0.25
Electroencephalogram (EEG)	362	(0.99)	472	(5.02)	834	(1.81)	< 0.001

**Table 3:** Tumor and clinical characteristics for 30-day readmissions.

**30-day readmissions**	**No epilepsy**		**Epilepsy**		**Total**		**p** **-value**
	**4000**	**(100.00)**	**1550**	**(100.00)**	**5550**	**(100.00)**	
Age 65 years							0.62
< 65 years	2220	(55.50)	839	(54.13)	3059	(55.12)	
≥ 65 years	1781	(44.53)	711	(45.87)	2491	(44.88)	
Gender							< 0.001
Male	1496	(37.40)	700	(45.16)	2196	(39.57)	
Female	2505	(62.63)	850	(54.84)	3355	(60.45)	
Charlson Comorbidity Index							0.01
None	1861	(46.53)	596	(38.45)	2457	(44.27)	
Score 1	1071	(26.78)	473	(30.52)	1545	(27.84)	
Score ≥ 2	1067	(26.68)	481	(31.03)	1548	(27.89)	
Frailty	310	(7.75)	121	(7.81)	431	(7.77)	0.96
Peritumoral cerebral edema	420	(10.50)	232	(14.97)	652	(11.75)	0.01
Hydrocephalus	268	(6.70)	98	(6.32)	366	(6.59)	0.80
Intracerebral hemorrhage	91	(2.26)	61	(3.96)	152	(2.74)	0.06
Brain compression	75	(1.88)	32	(2.06)	107	(1.93)	0.76
Fluid-electrolyte derangement	1182	(29.55)	431	(27.81)	1614	(29.08)	0.47
CNS infection (bacterial meningitis)	124	(3.10)	26	(1.68)	150	(2.70)	0.06
Steroid use	63	(1.58)	50	(3.23)	113	(2.04)	0.07

**Table 4:** Tumor and clinical characteristics for 90-day readmissions.

**90-day readmissions**	**No epilepsy**		**Epilepsy**		**Total**		**p** **-value**
	**7000**	**(100.00)**	**2637**	**(100.00)**	**9637**	**(100.00)**	
Age 65 years							0.08
< 65 years	3825	(54.64)	1344	(50.97)	5168	(53.63)	
≥ 65 years	3176	(45.37)	1293	(49.03)	4468	(46.36)	
Gender							0.04
Male	2620	(37.43)	1092	(41.41)	3713	(38.53)	
Female	4380	(62.57)	1544	(58.55)	5924	(61.47)	
Charlson Comorbidity Index							< 0.001
None	3134	(44.77)	993	(37.66)	4127	(42.82)	
Score 1	1789	(25.56)	734	(27.83)	2524	(26.19)	
Score ≥ 2	2077	(29.67)	909	(34.47)	2986	(30.98)	
Frailty	579	(8.27)	242	(9.18)	821	(8.52)	0.42
Peritumoral cerebral edema	584	(8.34)	377	(14.30)	961	(9.97)	< 0.001
Hydrocephalus	482	(6.89)	170	(6.45)	652	(6.77)	0.71
Intracerebral hemorrhage	111	(1.58)	77	(2.91)	188	(1.95)	0.02
Brain compression	146	(2.09)	61	(2.31)	207	(2.15)	0.67
Fluid-electrolyte derangement	1922	(27.46)	795	(30.15)	2717	(28.19)	0.16
CNS infection (bacterial meningitis)	163	(2.33)	36	(1.37)	199	(2.06)	0.05
Steroid use	117	(1.67)	73	(2.78)	190	(1.97)	0.09

**Table 5:** Duration from index discharges to 30- and 90-day readmissions.

	**Population**	**Mean ± standard deviation**	**Median (interquartile range)**	**Range**
30-day readmissions				
All 30-day readmissions	5550	12.30 ± 8.61	11 (4–19)	(1–30)
No epilepsy on readmission	4000	12.59 ± 8.60	11 (5–20)	(1–30)
Epilepsy on readmission	1550	11.52 ± 8.59	9 (4–18)	(1–30)
New onset	759	10.65 ± 8.47	8 (3–17)	(1–30)
Recurrent	791	12.39 ± 8.63	11 (4–19)	(1–30)
90-day readmissions				
All 90-day readmissions	9637	30.29 ± 25.03	23 (8–48)	(1–90)
No epilepsy on readmission	7000	30.19 ± 24.65	23 (9–47)	(1–90)
Epilepsy on readmission	2637	30.53 ± 26.03	23 (8–49)	(1–90)
New onset	1279	29.06 ± 25.80	21 (6–17)	(1–90)
Recurrent	1358	31.94 ± 26.20	24 (9–52)	(1–90)

*Note:* All units for mean, median, and range are in days.

**Table 6:** Multivariate analyses of postoperative epilepsy/seizures in 30-day and 90-day readmissions.

**Predictor variables**	**30-day readmissions**			**90-day readmissions**		
	**Odds ratios**	**95% confidence interval**	**p** **-value**	**Odds ratios**	**95% confidence interval**	**p** **-value**
Patient age ≥ 65 years	1.05	[0.96–1.15]	0.30	1.01	[0.94–1.08]	0.77
Male sex	1.58	[1.16–2.15]	< 0.001	1.22	[0.96–1.56]	0.10
Frailty	0.91	[0.58–1.42]	0.67	0.91	[0.66–1.27]	0.60
Grouped CCI score						
Score 1	1.56	[1.14–2.13]	0.01	1.35	[1.06–1.72]	0.02
Score ≥ 2	1.41	[1.04–1.92]	0.03	1.28	[1.02–1.59]	0.03
Preoperative epilepsy/seizure	8.15	[5.81–11.43]	< 0.001	8.36	[6.52–10.73]	< 0.001
Malignant meningioma	1.74	[1.12–2.69]	0.01	1.90	[1.39–2.61]	< 0.001
Peritumoral cerebral edema	1.46	[1.02–2.10]	0.04	1.78	[1.34–2.37]	< 0.001
Hydrocephalus	1.06	[0.53–2.12]	0.87	1.08	[0.65–1.78]	0.76
CVA/prior stroke	0.85	[0.53–1.37]	0.50	0.94	[0.64–1.37]	0.74
Brain compression	0.79	[0.55–1.15]	0.22	0.91	[0.68–1.22]	0.52
Fluid/electrolyte derangement	0.76	[0.58–1.09]	0.05	0.99	[0.81–1.22]	0.94
Bacterial meningitis	0.63	[0.32–1.24]	0.18	0.69	[0.39–1.24]	0.22
Steroid use	1.71	[0.68–4.29]	0.25	1.46	[0.77–2.80]	0.25
Intraoperative electrocorticography	0.31	[0.11–0.85]	0.02	0.21	[0.09–0.48]	< 0.001
*Hosmer–Lemeshow goodness of fit*	*F* = 0.21; Prob > *F* = 0.99			*F* = 0.97; Prob > *F* = 0.46		

## Data Availability

The data that support the findings of this study are available in Nationwide HCUP Databases at https://www.hcup-us.ahrq.gov/nrdoverview.jsp. These data were derived from the following resources available in the public domain: Nationwide Readmissions Database, https://hcup-us.ahrq.gov/db/nation/nrd/nrddbdocumentation.jsp
