# Supplementary material for: Predictors of Postoperative Epilepsy/Seizures in Patients Readmitted After Meningioma Resection
Source: Behav Neurol. 2025 Jul 9;2025:5717503. doi: 10.1155/bn/5717503 (PMC12267968; doi:10.1155/bn/5717503)
Supplement: Supporting Information — Additional supporting information can be found online in the Supporting Information section. Table S1: List of ICD-9 coding terminology utilized in data collection. [file 5717503.f1.docx]

**SUPPLEMENTARY TABLE 1: List of ICD 9 Coding terminology**

| **List of ICD-9-CM codes** |
| --- |
| Meningioma surgery: 0151,0159 |
| Epilepsy:345,780.39 |
| Meningioma: 225.2, 192.1, 237.6 |
| Peritumoral cerebral edema: 348.5 |
| Brain compression: 348.4 |
| Hydrocephalus:331.3-331.4 |
| Electrocorticography: 01.22-01.23 |
| Electroencephalography (EEG): 89.14, 89.19 |
| Placement of electrodes: 02.93 |
| CNS infection (bacterial meningitis): 320.0-320.9 |
| Fluid-electrolyte derangement: 276.0-276.9 |
| Spontaneous intracerebral hemorrhage: 431 |
